# Angioedema associated with dipeptidyl peptidase-IV inhibitors

**DOI:** 10.1186/s12948-021-00164-7

**Published:** 2021-12-06

**Authors:** Nicoletta Cassano, Eustachio Nettis, Elisabetta Di Leo, Francesca Ambrogio, Gino A. Vena, Caterina Foti

**Affiliations:** 1 Dermatology and Venereology Private Practice, Bari and Barletta, Italy; 2 Department of Emergency and Organ Transplantation, School and Chair of Allergy and Clinical Immunology, University of Bari “Aldo Moro”, Bari, Italy; 3 Section of Allergy and Clinical Immunology, Unit of Internal Medicine, “F. Miulli” Hospital, Strada Provinciale per Santeramo Km 4.100, Acquaviva delle Fonti, Bari, Italy; 4 Unit of Dermatology, Department of Biomedical Science and Human Oncology, University of Bari “Aldo Moro”, Bari, Italy

**Keywords:** Dipeptidyl peptidase-IV inhibitors, Gliptins, Angioedema, Angiotensin-converting enzyme inhibitors, Bradykinin

## Abstract

**Background:**

Dipeptidyl peptidase-IV (DPP-IV) inhibitors, also known as gliptins, are a class of oral antidiabetic agents. Postmarketing reports have documented the occurrence of angioedema in patients treated with gliptins and it was found that these drugs increased the risk of angioedema in patients concurrently treated with angiotensin-converting enzyme inhibitors (ACEIs). The aim of this manuscript is to provide an overview of the risk of angioedema associated with gliptins.

**Methods:**

The keywords used for the literature search in the PubMed database included “angioedema” and “dipeptidyl peptidase”, “gliptins”, or the name of each DPP-IV inhibitor. Articles in English published up to December 2020 were taken into consideration.

**Results:**

The available data appear to rule out a higher risk of angioedema associated with gliptin monotherapy and have revealed an increased susceptibility in patients simultaneously treated with gliptins and ACEIs. However, one single multicenter phase IV trial and case reports, even if very limited in number, have shown that angioedema can also occur during treatment with DPP-IV inhibitors without the concomitant use of ACEIs. The involvement of other drugs and drug interactions has occasionally been suggested. In a few patients, deficiency of enzymes involved in bradykinin catabolism was detected and this finding can constitute a risk factor for angioedema exacerbated by treatment with DPP-IV inhibitors.

**Conclusions:**

This risk of angioedema associated with the use of gliptins has mostly been related to the concurrent administration of ACEIs, and has been considered rare, but it might be underestimated and underreported. The role of additional risk factors or drug interactions deserves further investigations. Caution should be taken when considering the use of DPP-IV inhibitors in patients treated with ACEIs or presenting with other known risk factors for angioedema.

## Background

Dipeptidyl peptidase-IV (DPP-IV) inhibitors, also known as gliptins, are a class of oral agents available since 2006 for the treatment of type 2 diabetes mellitus that inhibit the degradation of incretins [1, 2]. DPP-IV is also involved in the degradation of other peptides, such as bradykinin and substance P [3–6], and gliptins could cause additional diabetes-unrelated effects through their action on further substrates.

Postmarketing reports have documented the occurrence of angioedema in patients treated with gliptins [7]. In particular, it was found that DPP-IV inhibitors increased the risk of angioedema in patients concurrently treated with angiotensin-converting enzyme (ACE) inhibitors (ACEIs) [5], a class of medications extensively used in clinical practice [8, 9].

Angioedema is a well-recognized side effect of ACEIs that has been estimated to affect 0.1–0.7% of exposed patients [6, 10]. The incidence of ACEI-associated angioedema may be underestimated in clinical trials because a history of angioedema is usually considered a strict exclusion criterion for ACEI therapy [8]. ACEI-induced angioedema has been shown to account for almost one-third of patients with acute angioedema presenting to emergency departments [11].

ACEI-induced angioedema most commonly involves lips, tongue, face, upper airways and rarely other sites, such as the abdomen [12], and can develop at any time during treatment with ACEIs, even after years from the initiation of therapy, suggesting that additional contributory factors may be implicated [6]. Several risk factors for the development of angioedema under ACEI therapy have been identified, such as ethnicity, with black and Hispanic patients at increased risk according to some reports [13–16]; history of either smoking or ACEI-induced cough [14]; history of drug allergy or seasonal allergies [16, 17]; concomitant use of calcium channel blockers, antihistamines and systemic corticosteroids [18] or nonsteroidal antiinflammatory drugs [16]; transplant and immunosuppressant use [19]; heart failure [8]. Age greater than 65 years [17, 18] and female sex [20] were identified as determinants of angioedema in ACEI-treated patients in some studies.

Angioedema due to ACEIs is believed to be the consequence of the decreased degradation of bradykinin and other vasoactive ACE substrates, such as substance P [3, 6].

ACE is a dipeptidyl-carboxypeptidase that converts angiotensin I to angiotensin II, a potent vasoconstrictor and an active component in the renin–angiotensin–aldosterone system, and has also a crucial role in the inactivation of the vasodilator bradykinin and substance P [21]. During ACE inhibition, the role of DPP-IV and other enzymes involved in the degradation of bradykinin and substance P becomes important [22, 23]. It has been supposed that genetic or environmental factors that reduce the activity of DPP-IV or other non-ACE enzyme pathways responsible for the inactivation of such vasoactive peptides are likely to augment the susceptibility to angioedema during exposure to ACEIs [6, 24]. DPP-IV protein concentration and activity were shown to be decreased in the sera of patients with ACEI-associated angioedema [25, 26]. Instead, diabetes mellitus has been defined as a protective factor against angioedema, and this finding was explained on the basis of the increased DPP-IV activity during hyperglycaemia [12, 27].

The aim of this manuscript is to provide an overview of the risk of angioedema associated with gliptins.

## Methodology

The keywords used for the literature search included “angioedema” and “dipeptidyl peptidase”, or “gliptins”, as well as the name of each DPP-IV inhibitor. Only articles in English were selected from the PubMed database. Clinical studies reporting angioedema as adverse event were included, as well as pharmacovigilance data and case reports. Articles from the references cited in the retrieved papers were also manually searched. The latest search was completed at the end of December 2020.

## Results of the literature search

The data from the retrieved publications were distinguished into two main categories. In the first part, safety information from randomized clinical trials and pharmacovigilance studies is presented. In the second part, detailed data extracted from single case reports were summarized. Brief comments obtained from the same literature were added, as appropriate.

## Clinical and safety studies

### Vildagliptin

In 2008, Brown et al. analysed the safety dataset of premarketing phase III randomized clinical trials with a duration up to 52 weeks in order to assess the incidence of angioedema in patients treated with vildagliptin versus those treated with comparators (placebo or antidiabetic drugs other than gliptins) [5]. The results of both the meta-analysis using the Peto method and the pooled analysis have shown no significant association between vildagliptin and angioedema overall. This observation was regarded as reassuring indicating that enzyme redundancy is generally sufficient to prevent accumulation of vasoactive peptides during DPP-IV inhibition [28]. Nevertheless, according to some authors [29], these findings should have been interpreted with caution because of the small sample size with insufficient statistical power, as the adverse reaction was rare.

However, in the same study of Brown et al. [5], among subjects taking an ACEI, vildagliptin users were shown to have an increased risk of angioedema [odds ratio (OR) 4.57, 95% confidence interval (CI) 1.57–13.28 in the meta-analysis; OR 9.29, 95% CI 1.22–70.70 in the pooled analysis)]. The risk of ACEI-associated angioedema was significantly increased among patients taking vildagliptin 100 mg/day, either in divided doses or as a single dose, but not in those treated with vildagliptin 50 mg/day, suggesting an apparent dose–response relationship. In most vildagliptin-treated patients, angioedema was judged to be mild or moderate in severity. The absolute risk was considered small [7], and the relatively modest impact of DPP-IV inhibition on the risk of ACEI-induced angioedema was ascribed to the presence of redundant enzymatic pathways implicated in the degradation of bradykinin and substance P [5]. Some limitations of the analysis by Brown et al. should be taken into account, as pointed out by the same authors [5]: (a) selection bias (most patients had been treated with an ACEI chronically prior to randomization, so that subjects at low risk for angioedema might have been selected); (b) lack of randomization in ACEI use within the vildagliptin and the comparator drug groups, so that it was not possible to compare angioedema rates in ACEI users versus non-users in the two groups; (c) limited (24-week to 52-week) duration of the phase III studies; (d) angioedema was non included among the primary endpoints of the phase III clinical trials. At the time of the publication written by Brown et al. [5], there were no sufficient data regarding DPP-IV inhibitors other than vildagliptin to conclude whether angioedema was to be considered a class effect or specific to vildagliptin [28].

A noninterventional cohort study explored the association of vildagliptin use with the risk of specific safety events of interest, including angioedema, compared with other noninsulin antidiabetic drugs using real-world data from five European electronic healthcare databases [30]. Patients with a previous angioedema event were not included in the analysis. Of the 738,054 adult patients with type 2 diabetes mellitus examined, 20,973 (2.8%) received vildagliptin with an average follow-up period of 1.4 years. No evidence was detected for an increased risk of angioedema associated with vildagliptin compared to other antidiabetic medications.

### Sitagliptin

Williams-Herman et al. reported no association of sitagliptin with “angioedema-related events”, regardless of concurrent ACEI exposure, in phase IIB and III trials up to 2 years in duration that recruited 6139 patients with type 2 diabetes receiving either sitagliptin 100 mg/day or placebo or an active comparator [31]. A subsequent analysis of 10,246 patients with type 2 diabetes who received either sitagliptin 100 mg/day or a comparator agent (placebo or an active comparator) in randomized, double-blind studies with 12-week to 106-week duration did not disclose a higher rate of angioedema or angioedema-related events in sitagliptin-treated patients compared to patients not exposed to sitagliptin, regardless of ACEI use [32]. Unfortunately, events were not adjudicated and the definition of “angioedema-related events” also comprised urticaria, anaphylactic and hypersensitivity reactions, thus confounding the interpretation of results [5].

### Saxagliptin

Among clinical trial recipients who received 2.5 or 5 mg saxagliptin daily, alone or in combination with metformin, a thiazolidinedione or glyburide, 1.5% had a “hypersensitivity-related event”, such as urticaria and facial angioedema, compared with 0.4% in the placebo recipients [7].

A post hoc analysis of data from 20 randomized, controlled studies in the phase IIb–IIIb clinical development programme as of 1 June 2011 was undertaken to evaluate the safety of saxagliptin as monotherapy or add-on therapy in patients with type 2 diabetes mellitus (n = 9156), focusing on events of special interest. In pooled datasets, the incidence rates of angioedema were comparable with saxagliptin and control (placebo or active comparator) [33].

SAVOR-TIMI 53 was a multicenter, randomized, double-blind, placebo-controlled, phase IV trial that allocated 16,492 patients with type 2 diabetes and risk for cardiovascular events to treatment with saxagliptin (at a daily dose of 5 mg or 2.5 mg in case of a glomerular filtration rate of ≤ 50 ml/min) or matching placebo (1:1 ratio). The median follow-up period was 2.1 years. There were no cases of fatal angioedema. Nonfatal angioedema occurred in 8 patients in the saxagliptin group and 1 in the placebo group (p = 0.04) [34]. It is worth mentioning that the number of patients recruited in the SAVOR-TIMI 53 trial treated with ACEIs was comparable in the saxagliptin and placebo groups. Therefore, this study was the only multicenter trial showing a significant increase in the rate of angioedema just associated with the use of saxagliptin, regardless of the concurrent treatment with ACEIs.

### Alogliptin

The EXAMINE trial was a phase III, multi-centre, randomized, double-blind, placebo-controlled study whose primary objective was to demonstrate the non-inferiority of alogliptin versus placebo with respect to major cardiovascular events. The study population consisted of 5380 patients, with 82% of them taking renin-angiotensin system blockers, although it was unknown how many patients received ACEIs. There was a similar incidence of “hypersensitivity” in the treatment and the control groups (0.6% and 0.5%, respectively), without any specific mention of angioedema cases. This study enrolled a selected group of patients with recent severe cardiovascular events as well as renal impairment and just over 20% of patients in both arms discontinued treatment, about 10% because of adverse events [29]. Serious adverse event reporting of patients enrolled in the EXAMINE trial (maximum time on study was 41 months) revealed the same frequency of angioedema (0.04%) in the alogliptin and placebo arms [35].

### Various agents

In the series of 84 patients with angioedema induced by renin–angiotensin–aldosterone system inhibitors described by Pfaue et al. [36], four patients were treated with both an ACEI and a DPP-IV inhibitor.

A recent disproportionality analysis of the World Health Organization’s pharmacovigilance database, VigiBase, examined the risk of angioedema associated with the combination of ACEIs and DPP-IV inhibitors compared with ACEIs alone or with DPP-IV inhibitors alone [3]. All reports of angioedema from November 14, 1967, to December 14, 2017 were extracted from VigiBase and all reports with symptoms suggesting histamine-mediated or hypersensitivity mechanisms were excluded in order to better identify bradykinin-mediated angioedema cases. The DPP-IV inhibitor drug class was involved in 677 reports of angioedema and 345 concerned ACEI and DPP-IV inhibitor combination (sitagliptin n = 225; linagliptin n = 37; vildagliptin n = 40; saxagliptin n = 43; alogliptin n = 3). No DPP-IV inhibitor was associated with an angioedema disproportionality signal, whereas the concomitant intake of ACEIs and DPP-IV inhibitors was associated with an increased reporting risk of angioedema [reporting OR 42.77 (95% CI 36.93–49.53)], suggesting a pharmacodynamic drug-drug interaction and a possible class effect [3].

## Case reports

Table 1 summarizes the most relevant data on case reports of angioedema related to DPP-IV inhibitors [37–48]. Different culprit agents were implicated and the role of either gliptin monotherapy or combination of DPP-IV inhibitors and other drugs was suspected.

**Table 1:** Case reports of angioedema related to DPP-IV inhibitors [37–48]

First author (reference)	Patient (sex, age)	Medical history (apart from diabetes mellitus)^a^	Treatments^b^	Clinical features of angioedema	Information on management and/or outcome of angioedema	Relevant investigations
Beaudouin [37]	56-year-old man	Prior acute coronary syndrome Dyslipidemia Seasonal rhinitis (not requiring treatment) Recurrent angioedema 5 years after the start of lisinopril, involving face, uvula and hands, on a yearly basis over 3–4 days, despite use of prednisolone	Sitagliptin 100 mg/day (last year) Lisinopril 10 mg/day (for 15 years) Glibenclamide, metformin, flurbiprofen, atorvastatin	After combination with sitagliptin, increased frequency of attacks (every 6 weeks) and association with abdominal pain	After withdrawal of lisinopril, disappearance of abdominal pain and occurrence of only 3 episodes of facial or foot swelling over 9 months After subsequent withdrawal of sitagliptin, absence of attacks (FU: 19 months)	Normal C1-INH level and function, and serum tryptase levels Sensitization to ragweed and grass pollens Deficiency of ACE, aminopeptidase P and carboxypeptidase N; DPP-IV results not available
Gabb [38]	66-year-old man	Hypertension Stable ischaemic heart disease Urolithiasis Benign prostatic hypertrophy Cough related to ramipril	Saxagliptin (last 6 months) Candesartan (for 5 years) Metformin, carvedilol, rosuvastatin	Foreign body sensation in the throat and dysphonia; oedema of the soft palate and uvula	Inefficacy of promethazine Discontinuation of candesartan	Normal complement levels
Gosmanov [39]	46-year-old African American woman	Uncomplicated hypertension Obesity Compensated vitamin D deficiency Riedel’s thyroiditis	Sitagliptin 50 mg + metformin 500 mg BID (last week) Losartan 100 mg/day Prednisone for thyroiditis	Upper and lower lip angioedema (concomitant pruritic morbilliform rash)	Discontinuation of sitagliptin, continuation of losartan and metformin monotherapy, and prednisone tapering, without any recurrence of symptoms	–
Hahn [40]	83-year-old woman	Hypertension Coronary heart disease	Saxagliptin 5 mg/day Ramipril 5 mg/day (for > 5 years)	Acute swelling of the tongue, followed by hypopharyngeal and supraglottical involvement	Interruption of ramipril but not of saxagliptin. No regression of symptoms within > 30 h despite various sequential treatments. Partial monitoring in an intensive care unit Initial use of IV prednisolone, IV antihistamine, inhalations with epinephrine, and then C1-INH. Subsequent intubation via the nasal route, and use of icatibant (initial improvement only after 8 h) Use of amlodipine instead of ramipril without any further attacks (FU:1.5 years)	Normal concentration of C4 and C1-INH Decreased ACE activity and increased C1-INH activity
Hermanrud [41]	Middle-aged Caucasian man	Hypertension	Daily use of sitagliptin Ramipril on a daily basis	Numerous episodes (47 in a year) in the head and neck area, occurring a few days after starting sitagliptin therapy Occasional severe lingual and laryngeal involvement	Referral to the ED in case of severe attacks. No effect of IV antihistamines and IV corticosteroids; no or sparse efficacy of epinephrine; some effect of C1-INH used once After ramipril discontinuation, severe oral, hypopharyngeal and laryngeal attacks with no response to high-dose antihistamines, steroids and epinephrine After substitution of sitagliptin with empagliflozin, only two episodes with slight swelling of the upper lip or tongue (FU: 12 months)	Complement and allergy tests with normal results
Millot [42]	67-year-old man	Hypertension Pulmonary embolism	Sitagliptin (last 2 months) Perindopril (for 10 years) Metformin, anti-vitamin K medication	Three episodes within 1 month; the third with significant glosso-pharyngo-laryngeal oedema and severe dyspnoea Two similar severe attacks in the following weeks	Regression in < 48 h of the first two attacks after use of antihistamines and corticosteroids Referral to ED for severe attacks For the third episode, supplemental oxygen, epinephrine aerosol, IV dexchlorpheniramine, IV methylprednisolone, without improvement in the next hour. After treatment with a concentrate of 4 coagulation factors used as an anti-vitamin K antagonist, regression of dyspnoea and dysphonia within 20 min, and of all symptoms within 8 h Resolution of the symptoms within 1 h from the use of C1-INH in the fourth episode, and within 30 min after injection of icatibant in the fifth	Histamine, tryptase, C3 and C4 levels, C1-INH level and function with normal results Negative auto-antibodies Low aminopeptidase P activity; no decreased activity of ACE and carboxypeptidase N
Saisho [43]	69-year-old man	Dyslipidaemia Gastroesophageal reflux disease	Vildagliptin 50 mg BID Ezetimibe, lansoprazole	Lower lip angioedema episodes occurring the day after starting vildagliptin therapy	Resolution after 1 day from vildagliptin interruption and switching to alogliptin 25 mg/day without recurrence (FU: 6 months)	–
Skalli [44]	79-year-old woman	History of major cardiovascular events Gastrointestinal disturbances with metformin	Sitagliptin Irbesartan 150 mg/day Insulin determir, atenolol, lysine acetylsalicylate, clopidogrel, atorvastatin, lansoprazole, clomipramine, hydroxyzine	Swelling of the lips, tongue and mouth, 14 days after starting sitagliptin	Remission within a few days after stopping sitagliptin After 10 days, reintroduction of sitagliptin and occurrence of angioedema with dyspnea 2 days later leading to permanent discontinuation of sitagliptin	–
Hamasaki [45]	60-year-old man	–	Anagliptin 200 mg/day	Severe edema of hands and face after a few weeks	Resolution at 2 weeks after the discontinuation of anagliptin	No detection of specific allergens
Arcani [46]	59-year-old man	Current cigarette smoking Two episodes of transient lip edema, several years earlier Recurrent angioedema in his daughter	Sitagliptin 50 mg/day (last 3 months) Metformin	Involvement of tongue, mouth floor, lower lip, right side of the face and neck, moderate dyspnea and dysphagia with sialorrhea	Referral to ED. No improvement after use of epinephrine aerosol, IV methylprednisolone, IV antihistamine, and then IV epinephrine. Subsequent IV administration of C1-INH and tranexamic acid with gradual improvement and resolution after 36 h No other episodes after discontinuation of sitagliptin (FU: 4 years)	Normal C1-INH levels and function, ACE levels, and aminopeptidase P activity Normal DPP-IV activity 1 week after stopping sitagliptin Reduced carboxypeptidase N activity Heterozygous Thr328Lys mutation in the coagulation factor XII gene
Schneider [47]	67-year-old Hispanic woman	Hypertension Hepatitis C virus infection Chronic renal failure Schizophrenia	Sitagliptin 100 mg/day Amlodipine, carvedilol, mirtazapine, olanzapine, insulin detemir (no changes to all these drugs in the past year) Glecaprevir/pibrentasvir started 2 weeks before her first attack	Two episodes separated by 1 month, involving the tongue with drooling and dyspnea In the second event, also edema of the lingual surface of the epiglottitis	Use of epinephrine and diphenhydramine in the first episode with resolution within a day. For the second event, referral to ED and use of epinephrine, dexamethasone, and diphenhydramine with slow improvement No further episodes after the discontinuation of sitagliptin (FU: 1 year), while remaining on glecaprevir/pibrentasvir for other 2 months	Normal C4 level during the event
Yeddi [48]	67-year-old African American man	–	Alogliptin 12.5 mg BID Metformin	Two weeks after starting alogliptin, facial, lip and tongue swelling on 4 consecutive mornings Few months later, re-treatment with alogliptin, and development of lip and face swelling within hours of ingestion of the first pill	Partial improvement with antihistamine for the initial episodes. Resolution over the next days following discontinuation of alogliptin In the last episode (after re-treatment), referral to ED. Gradual improvement with IV antihistamines and steroids, with resolution over the next 2 days No attacks after permanent discontinuation of alogliptin (FU: 6 months)	C4, C1-INH, and C1q binding assays within normal range

*ACE* angiotensin converting enzyme, *ACEI* angiotensin converting enzyme inhibitor, *BID* twice daily, *C1-INH* C1-esterase inhibitor, *DPP-IV* dipeptidyl peptidase‐IV, *ED* emergency department, *FU* follow-up period, *IV* intravenous

^a^All patients had type 2 diabetes mellitus; in the case described by Gosmanov and Fontenot diabetes was induced by corticosteroid therapy

^b^Dose and duration of treatment were reported for DPP-IV inhibitors and renin-angiotensin system inhibitors when information was available

In particular, angioedema was observed in four patients treated with ACEIs and gliptins [sitagliptin (n = 3) and saxagliptin (n = 1)] [37, 40–42] and recovered or markedly improved after discontinuation of both drugs [37, 41] or disappeared after the interruption of the ACEI alone [40], whereas in one case treatment changes were not specified [42]. In two of the cases managed with discontinuation of both drugs [37, 41], the ACEI alone was initially withdrawn but the attacks persisted even after this change. It is well known, however, that some patients with ACEI-induced angioedema may continue to have symptoms even after discontinuing ACEI treatment [12, 49]. Beaudouin et al. actually observed a patient with ACEI-related angioedema whose symptoms worsened after the addition of sitagliptin [37]. This patient was found to have a latent deficiency of bradykinin-degrading enzymes, supporting the relevance of the simultaneous ACE and DPP-IV inhibition in subjects with risk factors for angioedema.

Other authors described angioedema in patients concurrently treated with a DPP-IV inhibitor [sitagliptin (n = 2) and saxagliptin (n = 1)] and an angiotensin receptor blocker (ARB) [38, 39, 44]. The possible contributory role of the ARB led to discontinuation of the ARB in one case [38], whereas in other two patients the complete resolution of angioedema was achieved after stopping the DPP-IV inhibitor [39, 44]. The risk of angioedema due to ARBs is believed to be considerably lower in comparison with ACEIs [23]. A meta-analysis of randomized trials assessing the risk of angioedema with various renin-angiotensin system inhibitors has demonstrated that the overall weighted incidence of angioedema with ACEIs was 0.30% (95% CI 0.28 to 0.32) compared to 0.11% (95% CI 0.09 to 0.13) with ARBs and 0.07% with placebo (95% CI 0.05 to 0.09) [8]. However, the effect of combined treatment with gliptins and ARBs on angioedema has not specifically been investigated [38].

Angioedema attributed to gliptins alone was also recorded with different agents [sitagliptin (n = 1), vildagliptin (n = 1), anagliptin (n = 1), alogliptin (n = 1)] [43, 45, 46, 48]. Angioedema recovered after DPP-IV inhibitor discontinuation in three patients [45, 46, 48], and after the substitution of vildagliptin with alogliptin in a single report [43]. This last case might indicate that there is a difference in the risk of angioedema among gliptins, possibly linked to differences in the inhibition rate of DPP-IV among these drugs.

In the recent publication written by Schneider and Ramesh [47], angioedema took place in the setting of co-administration of glecaprevir/pibrentasvir for hepatitis C and sitagliptin. Because sitagliptin is a substrate of p-glycoprotein/ABCB1, which is inhibited by glecaprevir/pibrentasvir, the authors hypothesized that this drug interaction could give rise to increased serum concentrations of sitagliptin, thus inducing a more pronounced effect on DPP-IV inhibition and on the resulting mechanisms responsible for angioedema [47]. The temporal relationship of starting glecaprevir/pibrentasvir followed by the development of angioedema and the permanent recovery of symptoms after sitagliptin withdrawal might be hints supportive of the possible relevance of this drug interaction.

In the reported clinical cases, the influence of specific factors on the risk of angioedema cannot be ruled out, such as ethnicity [39, 47, 48], smoking [46], seasonal rhinitis [37], history of ACEI-related cough [38], use of prednisone for thyroiditis [39], flurbiprofen [37] or lysine acetylsalicylate [44]. Anyway, the limited number of case reports does not allow to gather sufficient information. The role of advanced age in conditioning the angioedema risk in patients treated with gliptins is not known. Interestingly, a decrease in the DPP-IV activity has been observed with increasing age [26].

Taking into account other details in case description, the absence of pruritus, urticaria or other symptoms suggesting histamine-mediated mechanisms was specified in various publications [37, 38, 40, 41, 43, 46–48]. On the contrary, Gosmanov and Fontanot observed the development of a pruritic morbilliform rash concomitantly with labial angioedema 1 week after starting an antidiabetic preparation containing sitagliptin [39].

The histaminergic nature of angioedema was also excluded in some reports on the basis of the lack of response to medications conventionally employed for the control of histamine-mediated and anaphylactic responses, including antihistamines, corticosteroids and/or epinephrine [38, 40–42, 46]. A gradual and slow improvement of symptoms following the use of similar medications was sometimes outlined [47, 48].

Some patients had severe symptoms, with hypopharyngeal and/or laryngeal oedema and dyspnea [40–42, 46, 47], and a few of them were ACEI users [40–42].

Drugs commonly used to treat bradykinin-mediated angioedema, such as the specific bradykinin B2-receptor antagonist icatibant and C1-esterase inhibitor (C1-INH) concentrate alone or with tranexamic acid, were sporadically required in severe refractory cases [40–42, 46]. Moreover, Millot et al. mentioned the occasional use of a four-factor prothrombin complex concentrate for anticoagulation reversal, in anticipation of intubation or tracheotomy, in a patient treated also with an anti-vitamin K agent, and they unexpectedly noted that this approach was effective in an angioedema episode [42].

Some reports contain information concerning laboratory investigations (Table 1), such as assessment of allergy [41, 45] or autoimmunity [42], C1-INH levels and function [37, 40, 42, 46, 48], complement factors [38, 40–42, 47, 48], or tryptase levels [37, 42], revealing normal results. It is important to underline that deficiency of enzymes involved in bradykinin catabolism was found in some patients [37, 42, 46]. This finding can constitute a risk factor for angioedema episodes exacerbated by DPP-IV inhibitor administration and was detected in a few patients with particularly severe symptoms [42, 46]. In one of these patients [46], type III hereditary angioedema was also diagnosed.

## Concluding remarks

The majority of data from clinical studies appear to rule out a higher risk of angioedema associated with the use of DPP-IV inhibitors as monotherapy, although the available information is still scarce. Moreover, most data were derived from randomized clinical trials that were not designed to assess specific adverse events. The ability to detect adverse events that are rare or very rare may be limited by the sample sizes, follow-up periods and other methodological features that generally characterize such trials.

The increased susceptibility to angioedema in patients simultaneously treated with gliptins and ACEIs, previously demonstrated by Brown et al. [5], has recently been confirmed by a pharmacovigilance data analysis, that has suggested a pharmacodynamic drug–drug interaction and a possible class effect [3].

Considering the wide simultaneous administration of DPP-IV inhibitors and ACEIs worldwide over the last years, angioedema has been considered rare as the literature contains only a few case reports [50]. Otherwise, it could be underestimated and underreported.

The case reports of gliptin-associated angioedema, even if limited in number, seem to suggest that this event can occur during treatment with DPP-IV inhibitors as monotherapy or combined with ACEIs, although the role of other drugs and additional risk factors cannot be excluded and deserves further investigations.

Angioedema can be life-threatening and challenging in its management. The available reports have shown a great variability in the severity of symptoms during angioedema attacks and in their response to various treatments. Anyway, the prompt discontinuation of the culprit drug is fundamental for the management of such reactions. Therefore, the development of angioedema in patients treated with gliptins should lead to the precautionary discontinuation of these drugs and concurrent culprit drugs, especially ACEIs.

Scott et al. published a comprehensive review focusing on the risk and pathomechanisms of angioedema due to inhibition of DPP-IV inhibitors as monotherapy and combined with ACEIs [29]. These authors stated that this risk might be overlooked in the clinical setting. They underlined that recent papers on large series of patients with angioedema did not mention DPP-IV inhibitors as possible trigger. Moreover, they encouraged an increased awareness of the risk of angioedema and implementation of reporting, and recommended that treatment with DPP-IV inhibitors must be carefully considered and monitored, especially in patients treated with ACEIs or with other known risk factors for angioedema.

## Data Availability

Not applicable.
